# Pilot study of a ketogenic diet in bipolar disorder

**DOI:** 10.1192/bjo.2023.568

**Published:** 2023-10-10

**Authors:** Nicole Needham, Iain H. Campbell, Helen Grossi, Ivana Kamenska, Benjamin P. Rigby, Sharon A. Simpson, Emma McIntosh, Pankaj Bahuguna, Ben Meadowcroft, Frances Creasy, Maja Mitchell-Grigorjeva, John Norrie, Gerard Thompson, Melissa C. Gibbs, Ailsa McLellan, Cheryl Fisher, Tessa Moses, Karl Burgess, Rachel Brown, Michael J. Thrippleton, Harry Campbell, Daniel J. Smith

**Affiliations:** Centre for Clinical Brain Sciences, University of Edinburgh, UK; Department of Nutrition and Dietetics, Royal Hospital for Children and Young People, NHS Lothian, UK; Usher Institute, University of Edinburgh, UK; School of Health and Wellbeing, University of Glasgow, UK; Health Economics and Health Technology Assessment, University of Glasgow, UK; Psychiatry, NHS Lothian, UK; Bipolar Scotland, UK; Department of Paediatric Neurology, Royal Hospital for Children and Young People, NHS Lothian, UK; Centre for Engineering Biology, University of Edinburgh, UK

**Keywords:** Bipolar type I or II disorders, clinical outcomes measures, other imaging, neurophysiology, metabolic psychiatry

## Abstract

**Background:**

Recent evidence from case reports suggests that a ketogenic diet may be effective for bipolar disorder. However, no clinical trials have been conducted to date.

**Aims:**

To assess the recruitment and feasibility of a ketogenic diet intervention in bipolar disorder.

**Method:**

Euthymic individuals with bipolar disorder were recruited to a 6–8 week trial of a modified ketogenic diet, and a range of clinical, economic and functional outcome measures were assessed. Study registration number: ISRCTN61613198.

**Results:**

Of 27 recruited participants, 26 commenced and 20 completed the modified ketogenic diet for 6–8 weeks. The outcomes data-set was 95% complete for daily ketone measures, 95% complete for daily glucose measures and 95% complete for daily ecological momentary assessment of symptoms during the intervention period. Mean daily blood ketone readings were 1.3 mmol/L (s.d. = 0.77, median = 1.1) during the intervention period, and 91% of all readings indicated ketosis, suggesting a high degree of adherence to the diet. Over 91% of daily blood glucose readings were within normal range, with 9% indicating mild hypoglycaemia. Eleven minor adverse events were recorded, including fatigue, constipation, drowsiness and hunger. One serious adverse event was reported (euglycemic ketoacidosis in a participant taking SGLT2-inhibitor medication).

**Conclusions:**

The recruitment and retention of euthymic individuals with bipolar disorder to a 6–8 week ketogenic diet intervention was feasible, with high completion rates for outcome measures. The majority of participants reached and maintained ketosis, and adverse events were generally mild and modifiable. A future randomised controlled trial is now warranted.

Bipolar disorder is a serious mental illness with a lifetime risk of 1–2%.^1^ The global burden of bipolar disorder, measured in disability-adjusted life years, rose by 14.9% over the period 2005–2015.^2^ It usually begins in early adulthood and has considerable economic and societal impact. However, the underlying pathophysiology remains poorly understood, and current treatment and prevention strategies are suboptimal, with a need to identify effective new interventions.

Abnormal glucose metabolism is common in people with bipolar disorder, with an increased prevalence of insulin resistance and type 2 diabetes.^3,4^ It is possible that hyperinsulinemia may adversely affect the production of energy by mitochondria in the brain.^5–7^ Ketones can provide an alternative energy source to glucose, which may bypass this process and allow for more stable variations in energy production.^8^

The ketogenic diet is a safe and effective therapy for seizure reduction in children with drug-resistant epilepsy, as evidenced by a Cochrane review of randomised controlled trials and almost a century of clinical use.^9,10^ The diet's most significant effect is that it induces ketosis, wherein mitochondria in the body and brain preferentially oxidise fatty acids to produce ketones (beta-hydroxybutyrate and acetoacetate) and then use these as a source of energy (instead of glucose). This metabolic shift reduces seizures by 50% in around half of children with drug-resistant epilepsy.^11^ There are clinical parallels between bipolar disorder and epilepsy, such as the pharmacological treatments that are effective, which could indicate similarities in underlying disease processes.^12^

Case series have suggested benefits of a ketogenic diet in several psychiatric disorders, including bipolar disorder^13,14^ and schizophrenia.^15,16^ Analysis of data from online bipolar disorder forums (165 people with bipolar disorder adhering to a ketogenic diet) found that 56% reported either remission of symptoms or significant mood stabilisation.^4^ Recent narrative reviews have indicated that a ketogenic diet may affect metabolic and biochemical features of bipolar disorder, including: reduction in oxidative stress; improved mitochondrial function and biogenesis; improved glutamate/GABA transmission; and reductions in intracellular sodium and calcium.^17^

In this pilot study, we aimed to assess recruitment, acceptability and feasibility of the intervention and completion of relevant outcome measures. Secondary objectives (to be reported in a separate publication) were to assess the relationships of biochemical, metabolomic and brain imaging biomarkers with clinical and functional outcomes.

## Method

### Design, approvals and consent

This was a single-group non-randomised interventional pilot study with no control arm. The authors assert that all procedures contributing to this work comply with the ethical standards of the relevant national and institutional committees on human experimentation and with the Helsinki Declaration of 1975, as revised in 2008. The study received a favourable ethical opinion from the South East Scotland Research Ethics Committee 02 (approval number 22/SS/0007) and was approved by NHS Lothian Research and Development. Sponsorship was provided by the Academic and Clinical Central Office for Research and Development. Written informed consent was obtained from all participants. The study was prospectively registered at isrctn.com under registration number ISRCTN61613198 on 2 March 2022.

### Study participants

Participants were recruited from 27 April 2022 through Bipolar Scotland via email circulars, local groups and targeted social media. Eligible participants were those with a diagnosis of bipolar disorder, as per the DSM-IV diagnostic criteria, who had been clinically euthymic for 3 months (defined as no episodes of depression or hypomania/mania). They had to be aged 18–70 years, with the upper age limit set to improve the comparability of magnetic resonance (MR) head scans. Participants needed to have a sufficient understanding of English and to be based in Scotland. Initial exclusion criteria included: pregnancy, breastfeeding or planning to become pregnant within 3 months; active substance misuse; use of a ketogenic diet within 2 months; following a vegan diet; admission to hospital within 3 months; involvement in any other research; inability to complete baseline assessments; liver, kidney, or cardiovascular disease; and severe hyperlipidaemia (familial hypercholesterolaemia or total cholesterol >7.5 mmol/L). The following exclusion criteria were added during the study period: diabetes; training for or undertaking very high-energy-requirement activities; and significant changes to psychotropic medication, planned or within 3 months. Reasons for these additional exclusion criteria are described below.

### Participant assessments and data collection

Pre-recruitment appointments included an explanation of the study to potential participants and checking of eligibility criteria. At baseline appointments, participants received detailed information about establishing and maintaining a ketogenic diet, including potential risks and instructions on how to complete monitoring. They were asked to complete and return a 3 day food diary and a pre-ketogenic-diet information sheet beforehand to aid planning and personalisation of the diet.

At baseline and at 6–8 week follow-up assessments, medical and medication history were reviewed, blood pressure and body mass index (BMI) were measured, and a diagnostic interview was completed. Participants completed a range of mental health measures (Affective Lability Scale 18; Beck's Depression Inventory; Young Mania Rating Scale). Quality of life measures included the Within Trial Resource Use Questionnaire, developed to identify key health and social care resource use in addition to personal household expenditure on food and beverages and employment/absenteeism information; the EuroQol 5D quality of life instrument;^18^ and the Work Productivity and Activity Impairment Questionnaire (tailored). Fasting venepuncture and MR brain scans were completed before and after the intervention. Questionnaires were either completed on paper or later entered into a secure online platform by participants. Face-to-face contacts took place at the Clinical Research Facility and at the Edinburgh Imaging Facility at the Royal Infirmary of Edinburgh.

Participants were asked to take daily capillary readings of glucose and ketones on a KetoMojo device. Initial participants texted daily readings to the research team, but they and later participants were subsequently able to sync readings via Bluetooth to an app on their phone and provide the research team with permission to access the results via an online platform. Participants were also asked to complete daily ecological momentary assessments (EMAs) of anxiety, mood, energy, impulsivity and speed of thought. These were initially sent by text, with an online data collection tool trialled for the final two participants. Participants were asked to complete continuous accelerometry for a period of 9 weeks, using three consecutively worn *AX3* actigraph devices for 3 weeks each.

All participants were offered weekly remote contact with a dietitian during the intervention period, with additional contacts as required. They had the contact details of a psychiatrist on the research team, who was available to address concerns about their mental state or other aspects of the study.

Post-intervention, 15 participants and four health professionals who were involved in the study took part in a semi-structured interview as part of a process evaluation (to be reported separately).

### Intervention

A modified ketogenic diet was used, with estimated energy requirements of approximately 60–75% from fat, 5–7% from carbohydrate and additional calories from protein.^11^ Unsaturated fats were encouraged to minimise the risk of increased blood cholesterol and triglycerides. Individual macronutrient percentages were altered over time based on a range of factors, including levels of ketosis. Ketosis is achieved most easily if participants ingest as near to their daily energy requirements as possible. Where weight loss was desired and safe, an energy deficit was prescribed to encourage the use of body fat as an additional ketone source. Participants were established on the intervention for a 6–8 week period, including a brief adaptation period. Medical treatment from regular care teams continued as standard. The modified ketogenic diet information sheet specified desirable blood ketone levels as 1–4 mmol/L and glucose levels as 4–7.8 mmol/L.

Support during the intervention period included confirming participants’ understanding of the intervention, supporting adherence to the diet, problem-solving, and identifying and managing side-effects. Individual dietary prescriptions included the total number of calories and grams of macronutrients per day, which were divided into portions. Recipes were adapted for individual dietary prescriptions for each participant. Participants were supervised during the dietary cessation period or given further support if they chose to continue with the diet. A behavioural component to support the likelihood of adherence to the diet was incorporated, using fortnightly fidelity checklists. This was informed by the COM-B framework,^19^ which asserts that behaviours (in this case, adherence) are influenced by three main factors: capability, opportunity and motivation. The intervention addressed these elements and drew on theories including social cognitive theory and self-determination theory.

In line with international recommendations,^20^ and given that a ketogenic diet increases the risk of inadequate micronutrient intake,^21^ participants were advised to increase their fluid consumption and to take an easily available Tesco A-Z multivitamin and mineral supplement, and a Tesco Calcium and Vitamin D supplement. The modified ketogenic diet information sheet described how to manage adverse events, e.g. hypoglycaemia or hyperketosis. It was necessary for all participants to have urea and electrolytes analysis, liver function tests and a lipid profile prior to commencing the diet and at 6–8 week follow-up to exclude significant hepatic or renal dysfunction or familial hypercholesterolaemia and to identify possible adverse changes associated with the diet, such as changes in liver function or lipid profile.

### Primary and secondary outcomes

The primary outcomes of interest were participant adherence to the ketogenic diet during the study period, completion of weekly dietitian interviews, and completeness of daily ketone levels, glucose levels and EMA data. Staff time, attrition rate and details of side-effects were also measured, and a health economics analysis was completed. A more detailed process evaluation and evaluation of the behavioural component of the intervention was carried out and will be reported separately. Detailed data on a range of secondary outcomes will also be described in a separate publication, including symptom questionnaires, physical health parameters, actigraphy, EMA data, HbA1c, C-reactive protein, beta-hydroxybutyrate, insulin levels, fasting glucose levels, and serum and brain MR measures of metabolites.

### Analysis

Descriptive statistics were used for recruitment, retention and outcome data completion rates. Analyses of ketone and glucose readings were performed using Excel and SPSS with appropriate measures of central tendency, frequency and dispersion. Levels of ketosis were stratified according to the KetoMojo user instructions: ‘light ketosis’ was defined as 0.5–1.0 mmol/L, ‘optimal ketosis’ as 1–3 mmol/L and ‘high ketosis’ as 3–5 mmol/L. Three levels of hypoglycaemia were identified:^22^ mild (level 1; 3.0–3.9 mmol/L), moderate (level 2; 2.2–2.9 mmol/L) and high (level 3; <2.1 mmol/L), and percentages of occurrences were calculated.

## Results

### Sample description

**Fig. 1:**
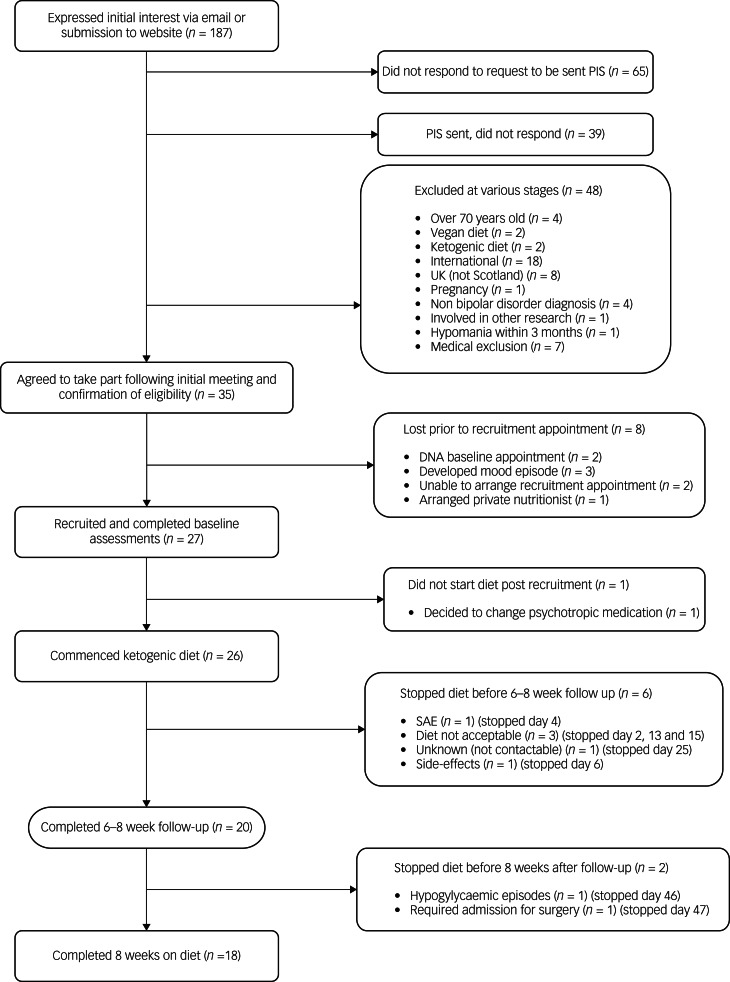
Flow chart illustrating the recruitment and completion rates of participants in the study. PIS, participant information sheet; SAE, serious adverse event.

Figure 1 summarises recruitment and retention. Recruitment took place over 37 weeks, with 27 participants recruited and 26 commencing the diet. Of the 26 recruited, eight (31%) were men and 18 (69%) were women, aged between 26 and 54 years (mean age 45 years). Twenty-five met criteria for type I bipolar disorder, and one met the criteria for type II bipolar disorder. Eight participants were within the normal BMI range, nine were overweight and nine were obese. Sixteen participants were prescribed an antipsychotic, 16 were prescribed a mood stabiliser and nine were prescribed an antidepressant. Ten participants had a combination of two psychotropic classes and four a combination of all three classes. Three participants were not prescribed any psychotropic medication. Participants were from a wide geographical spread across Scotland: Aberdeen City (*n* = 2); Aberdeenshire (*n* = 1); Dumfries and Galloway (*n* = 1); Dundee City (*n* = 1); East Ayrshire (*n* = 1); East Lothian (*n* = 2); Edinburgh (city of) (*n* = 8); Fife (*n* = 1); Glasgow City (*n* = 3); Highland (*n* = 2); North Lanarkshire (*n* = 2); Scottish Borders (*n* = 1); and West Lothian (*n* = 1).

Of 26 baseline appointments, 23 were in person and three were online. For the 20 follow-up appointments, 12 were in person and eight were online, excluding fasting bloods and MR scans. Two participants completed both baseline and follow-up appointments online. Twenty participants followed the diet until their 6–8 week follow-up appointment, and 18 did so for the full 8 week intervention period. All additional contacts with the dietitian were remote, and no requests for face-to-face meetings were made. All aspects of the study could be managed remotely, with no safety compromises identified.

Of the five participants who chose to discontinue the diet prior to follow-up, the reasons were as follows: insomnia after transitioning very quickly, against dietitian advice, with additional indigestion, nausea and migraine, and concerns about developing a depressive episode, and difficulties with the change of routine for family (discontinued day 2); side-effects including feeling very grumpy and hungry (day 6); difficulty following the dietary plan (discontinued day 13); discomfort around dietary monitoring, including weekly body weight measurement and daily glucose levels, and concerns about the possibility of triggering disordered eating patterns and apprehensiveness regarding food intake (discontinued day 15); unknown as uncontactable from day 25.

### Data completion

Of the 20 participants who completed the intervention, 100% completed both baseline and follow-up appointments and questionnaires; 19 completed baseline and follow-up weight, height and blood pressure; 18 completed baseline and follow-up fasting MR scans; and 17 completed baseline and follow-up fasting blood tests. Reasons for missing assessments included one participant forgetting to fast prior to initial bloods and their MR scan, one unsuccessful phlebotomy and one person not feeling well enough to attend follow-up in person. The median time from completion of baseline appointments to starting the diet was 6 days (some participants delayed commencing the diet for practical reasons).

During the intervention period, 95% of both daily ketone and glucose measures and 93% of daily EMA measures were provided by participants. In the 2 week post-intervention period, this dropped to 66% for ketones, 64% for glucose and 52% for EMA data.

Forty-nine of 60 (81.7%) actigraph devices given to the 20 participants were returned with data recorded on them, with a variety of practical difficulties responsible for the missing or blank devices. The median number of days of data collected from each viable actigraph was 19 (minimum 6 days and maximum 20 days), meaning that 822 of a planned 1260 (65%) days of accelerometry data were collected. One participant required a new actigraph strap as it snapped, and another reported a skin reaction to the strap, both reducing wear time. Other difficulties included postal strikes delaying participants receiving the devices.

### Physiological results

Of 45 sets of baseline and follow-up blood samples from all participants, 34 demonstrated at least one parameter outside the normal laboratory reference range that was relayed to the primary care team, the majority relating to a non-urgent altered lipid profile. All but one participant for whom baseline and follow-up data were completed (*n* = 20) lost weight during the intervention period. The median weight change was a loss of 4.5 kg (9.9 lbs), with weight loss ranging from 0.2 to 10 kg. Significant weight loss (≥5% body weight) was achieved by 52.6% of participants. Weight loss was supervised by the dietitian and was intentional in those who were overweight or obese at recruitment and desired weight loss. Of 16 participants with baseline and follow-up fasting beta-hydroxybutyrate levels available, mean baseline levels were 0.10 ± 0.02 mmol/L (min = 0.05, max = 0.14, median = 0.11), and follow-up levels were 0.88 ± 0.99 mmol/L (min = 0.10, max = 3.70, median = 0.49). Three of the six participants who discontinued the diet had raised baseline beta-hydroxybutyrate levels (>0.1) of 0.13, 0.68 (participant known to fast) and 0.82 (participant who had type 2 diabetes and developed ketoacidosis).

### Daily ketone and glucose levels

**Fig. 2:**
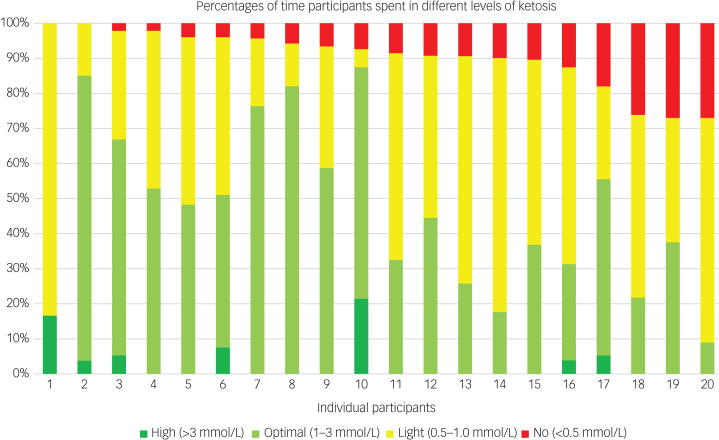
Percentages of daily participant readings representing different levels of ketosis over a 6–8 week period. Participants are ordered from highest percentage of total days with a ketone level indicating ketosis (>0.5 mmol/L) to lowest. Days where data were missing were excluded from the analysis.

The following results refer to the available ketone levels in 20 participants who continued a modified ketogenic diet for 6–8 weeks and who completed follow-up assessments. All 20 participants achieved daily readings indicating light ketosis (0.5–1.0 mmol/L) within 1–7 days of commencing a ketogenic diet and optimal ketosis (1–3 mmol/L) within 3–13 days. Of all the readings available, 91% indicated at least light ketosis (>0.5 mmol/L). Participants’ percentages of daily readings indicating light ketosis or above ranged from 73 to 100% (Fig. 2). Overall mean daily ketone levels were 1.3 mmol/L (median = 1.1 mmol/L). From daily readings, the percentage indicative of ketosis was over 70% for all 20 participants (≥80% for 17 participants, ≥90% for 15 participants and 100% for two participants). The majority of daily glucose readings (91%) were in the normal range (4.0–7.8 mmol/L), with 9% suggesting mild hypoglycaemia (3.0–3.9 mmol/L).

### Dietary changes during intervention

The study dietitian adapted and personalised the dietary prescription for each participant throughout the study. Thirty-two dietary prescription changes were required during the intervention, ranging from 0–5 per participant (median = 1). The most frequent reasons for prescription changes were: the participant's preference regarding the number or size of food portions; the need to enhance ketone production; achieving prespecified loss of weight; and addressing unintended weight loss. The median dietitian time required to support each participant was 505 min, excluding the baseline meeting. Nine participants (45%) required a decrease in calories, ranging from 100–200 kcals, on 1–4 occasions during the intervention period, as their total energy requirements had decreased following weight loss. Three participants (15%) required additional fat to promote further ketosis. Owing to follow-up changes in lipid profile, including an increase in total cholesterol, low-density lipoprotein (LDL) cholesterol or triglycerides, six participants (30%) were advised to increase the amount of omega-3 fatty acids in their diet and to reduce saturated fat intake where possible.

### Side-effects

Table 1 shows the numbers and proportions of participants reporting known side-effects from a ketogenic diet; these side-effects were generally mild and resolved with dietary prescription changes. One participant had 14 incidents of mild hypoglycaemia (ten reported routinely via daily readings, four reported additionally), which started on day 24 of the intervention and were frequently associated with symptoms of hypoglycaemia. Their only regular medication was gabapentin. These continued despite six changes to their dietary prescription, which led to the intervention period being shortened.

**Table 1:** Side-effects reported during the 6–8 week intervention period. Data are for 20 participants completing the intervention period

Side-effect	Number of participants experiencing the side-effect	Percentage of participants experiencing the side-effect (%)	Number of weeks side-effect reported per participant (mean)	Number of weeks side-effect reported per participant (median)
Fatigue	12	60	2.2	2.0
Constipation	11	55	1.1	1.0
Drowsiness	10	50	1.2	1.0
Hunger	10	50	1.6	1.0
Thirst	8	40	2.7	2.5
Irritability	8	40	1.7	1.5
Diarrhoea	6	30	0.6	0.5
Hypoglycaemia	4	20	0.6	0.0
Nausea	2	10	0.3	0.0
Vomiting	2	10	0.2	0.0
Excess ketosis	1	5	0.0	0.0

### Serious adverse events

One participant reported ketone levels of 7.1 mmol/L on day 3 of the intervention, with associated fatigue and nausea, and required hospital admission for treatment of euglycemic ketoacidosis (with intravenous insulin and glucose), which resolved after 2 h of treatment. This participant had type II diabetes and was prescribed empagliflozin and pioglitazone. The medical team who treated the participant in hospital concluded that this outcome was likely to have been the result of combining SGLT2 inhibitors with the initiation of a ketogenic diet. Following treatment, this participant recovered quickly and was subsequently withdrawn from the study.

### Barriers to following a ketogenic diet

Process evaluation and experiences of the study team identified some potential barriers when following a ketogenic diet, including difficulty with the organisation required for food shopping and meal preparation, the affordability of food, inconvenience around the timing of meals, and increased satiety owing to high fat intake. One participant had a lactose intolerance, which limited their food choices on the diet. Other process evaluation feedback included the positive impact of weight loss and improvements in energy, mood and self-control, as well as trying new foods.

### Medical input

Of the 20 participants who completed follow-up, one required a reduction in their fluoxetine by their community psychiatrist following a 1 month period of hypomania during the study. This had not fully resolved by the end of the study, but the participant had good insight into this and was continuing to work. There was one reported episode of hypomania within a week of discontinuing the diet, which lasted 3–4 weeks and self-resolved. One participant developed a depressive episode during the intervention period, which resolved before their follow-up appointment. All study participants had some contact with a psychiatrist on the study team (N.N.) during follow-up, mostly to discuss administrative tasks, abnormal blood results, and physical and mental health symptoms. The median contact time per participant was 18 min (mean = 26).

### Health economics

Twenty participants completed all baseline and follow-up health economics questionnaires, with minimal missing data (<3%). Forty-four per cent of the sample were employed at baseline, and 50% were employed at follow-up. The percentages of participants reporting ‘no problems’ for each attribute in the EQ5D-5L at baseline and follow-up, respectively, were: mobility, 90 and 85%; self-care, 90 and 85%; usual activities, 65 and 55%; pain and discomfort, 45 and 45%; and anxiety and depression, 45 and 50%. One participant reported level 5 (severe) for pain. The visual analogue scale (VAS) utility scores at baseline and follow up were 66.7 (CI: 58.9–74.4) and 64.2 (CI: 55.0–73.4), respectively. Of the 20 participants, almost 50% reported the effect of sickness on their work productivity and routine work. Mean productivity loss of 1.9 h (CI: 0.83–3.00) in the last week was reported at baseline and 2.4 h (CI: 0.86–4.25) at follow-up. At baseline, mean expenditure of £14 per week (CI: 7.4–20) was reported on take-away meals, compared with £4.7 (CI: 0.1–9.2) at follow-up. Resource use was most frequently reported for general practitioner and practice nurse visits.

## Discussion

### Recruitment, retention and mode of delivery

Overall, we found that recruiting people with bipolar disorder to a ketogenic diet intervention was feasible. Recruitment via Bipolar Scotland was successful for this pilot and could be extended to other UK-wide support groups, as well as direct recruitment through National Health Service clinics, for a future randomised controlled trial. Given the episodic nature of bipolar disorder, the partial loss of participants between initial and baseline meetings was expected. We also found that the majority of participants found the intervention acceptable and could be safely established and followed up using mostly remote consultations. This approach has not been tested in other ketogenic diet studies and could facilitate recruitment from a wide geographic area in future studies.

Several additional exclusion criteria were identified during this pilot study. People with diabetes were excluded owing to safety concerns after a serious adverse event in a participant taking an SLGT-2 inhibitor. This approach was cautious, and with an improved understanding of the increased risk of ketoacidosis specifically in people taking SGLT2 inhibitors (and following a ketogenic diet, a predisposing factor for developing this),^23^ future recommendations would be to only exclude people with type II diabetes prescribed this medication class. People undertaking high-energy intensive exercise were excluded owing to concerns about feasibility of the diet. One participant who withdrew as they found the diet unacceptable was engaged in prior intermittent fasting and had a raised baseline hydroxybutyrate level – future trials should consider excluding participants who regularly fast, as they may already have increased ketone production. It is also important for researchers to have access to participant medical records during recruitment to improve the accuracy and reliability of medical information, including comorbid diagnoses.

The study withdrawal rate of 23% was comparable with those of other pilot studies of ketogenic diet in various conditions, ranging from 15% in recurrent glioblastoma^24^ and 18% in Parkinson's disease^25^ to 35% in advanced malignancies.^26^ The majority of withdrawals were due to difficulties with aspects of the diet (including the intensive monitoring) and not following advice to initiate gradually. To improve retention rates in a future trial, more intensive dietitian input pre-recruitment may help to increase participant understanding of what the diet and monitoring entails, including the importance of starting slowly to reduce the risk of side-effects. Potential recruits could also be provided with example recipe sheets for sample ketogenic meals in advance and more detailed information on food costs. The opportunity to join group sessions before and during the study and some element of social support may also help retention.^27^ A more detailed qualitative assessment of participant experiences during the study will be reported separately. The median time input of 18 min (mean = 26) required from a psychiatrist highlights that if adequate dietitian input is available, the clinical input required from a psychiatrist is likely be minimal in euthymic patients.

We found very high completion rates of all baseline assessments, including fasting MR scans and blood tests. One participant forgot to fast (future trials should check this before completing fasting assessments and allow rescheduling). Despite participants needing to complete EMA data via a texting system, the overall compliance was high. Submission of ketone, glucose and EMA data was high during the intervention period but might be improved by reminders provided via a mobile app. Difficulties with the completion of follow-up bloods or MR scans were mostly due to unforeseen participant circumstances. The impact of this could be reduced by increasing capacity for rearranging blood and imaging appointments and by issuing reminders about prior fasting. The utility of the actigraphs could be improved by using more advanced technology. This could include devices with a battery life to last the entire intervention or the ability to upload data remotely in real time. Further education regarding the benefits of collecting actigraph data may also improve compliance.

### Ketone levels and adherence

Adherence to a ketogenic diet can be challenging, and it may be expected to be especially so in individuals with bipolar disorder, where depressive symptoms can have a negative impact on motivation.^8^ However, we found that despite the barriers identified, adherence to a ketogenic diet was high, with 91% of daily readings indicating ketosis. This was probably related to the continuous support from a specialist dietitian using an individualised and adaptable approach. All of the participants were in ketosis at least 70% of the time. Weekly ketone levels from finger-prick measurements showed a mean of 1.3 ± 0.8 and median of 1.1 mmol/L. These levels are similar to those reported in other ketogenic diet studies, including in Parkinson's disease (mean daily blood ketone levels 1.15 ± 0.59 mmol/L^25^) and Alzheimer's disease (mean weekly blood ketone levels 0.95 ± 0.34 mmol/L^28^).

There is no gold standard to measure adherence in dietary interventions, but for a ketogenic diet, blood ketone measurements are relatively easy and specific and are more accurate than urinary levels.^25^ Ketone levels fluctuate throughout the day, limiting the interpretation of once-daily ketone measures. Continuous ketone monitoring devices may improve the accuracy of results and provide a day-to-day measure of adherence. In future controlled trials, other self-reported measures of adherence such as 24 h food recalls or nutritional questionnaires could be incorporated to better understand the challenges of this dietary intervention. The production and maintenance of ketones may differ across individuals, and methods to record food intake beyond biomarker recording should increase our understanding of the barriers to achieving continuous ketosis.^29^

### Side-effects and adverse events

Side-effects were common and typically mild and could usually be resolved with dietary adjustments such as increased fibre content and fluid intake for constipation.^20^ These were uncontrolled observations and so it was not possible to determine whether each adverse event was directly related to the intervention. Although there were changes in lipid profiles (increased levels of total cholesterol, LDL or triglycerides) at follow-up in nine participants, this was expected, and such changes usually return to normal limits over time.^30,31^ Four participants had one or more incidents of hypoglycaemia (<4 mmol/L), which resolved with dietary changes. This highlights a need for regular monitoring, which could be done more accurately and safely with continuous glucose monitoring. One participant had persistent mild but symptomatic hypoglycaemia and struggled to maintain a blood glucose of >4 mmol/L despite many dietary changes, indicating the need to consider stopping the diet if this occurs.

The serious adverse event in the form of euglycemic ketoacidosis during dietary initiation was a novel finding. Other trials using a ketogenic diet as an intervention have started excluding participants on SGLT2 inhibitors; this will be important for future studies.

### Health economics

Health economic instruments were completed with minimal missing data, indicating their feasibility for future trials in this population. The EQ5D-5L VAS mean score of 64–68 could be benchmarked against population values of 83–85 (depending upon the source used), indicating a lower quality of life in this participant group. The findings from the health economic data are indicative of the level and variability of all reported values, possible changes in baseline levels of expenditures, quality of life and productivity at follow up; and of the applicability of using such health economic instruments in larger-scale studies.

### Strengths and limitations

The strengths of our study include the relatively high completion of baseline and follow-up assessments, including detailed resource use and quality of life instruments required for any future cost-effectiveness analyses; high completeness of the ketone, glucose and EMA readings; successful induction and maintenance of ketosis; and effective delivery of dietetic support. The identification of new exclusion criteria will improve the safety and efficacy of future trials. Despite the barriers identified, 77% of participants remained on the diet until follow-up, consistent with other ketogenic diet research.^32^

The nature of the ketogenic diet used required a cohort of participants with high levels of motivation, commitment and time. This may limit the applicability of this intervention to the wider bipolar disorder population. We also limited our study to people who were currently euthymic. In the future it would be helpful to assess the feasibility of this intervention for bipolar depression.

### Clinical implications

This pilot study is one of the first to explore the feasibility and safety of a ketogenic diet in people with bipolar disorder. We provide evidence that this intervention is feasible and safe. Moreover, it is clear that people with bipolar disorder are open to considering dietary/nutritional interventions as adjunctive therapies, and that the predominantly remote recruitment and conduct of a ketogenic diet intervention are possible. Our study also showed that a future trial could readily identify and measure all the resource use, quality of life, and wider household and productivity impacts relevant for conducting a full cost-effectiveness/cost-utility analysis. We conclude that the next step should be a randomised controlled trial of a ketogenic diet in bipolar disorder.

## Data Availability

The data that support the findings of this study are not publicly available as explicit consent was not sought from participants.
